# Endometrial Cultures in Acute Pelvic Inflammatory Disease

**DOI:** 10.1155/S1064744995000317

**Published:** 1995

**Authors:** Soheil Amin-Hanjani, Ashwin Chatwani

**Affiliations:** 1 Department of Obstetrics and Gynecology Goddard Medical Associates, P. C. Brockton MA USA; 2 Department of Obstetrics and Gynecology Temple University Hospital 3401 N. Broad Street 7OPD Philadelphia PA 19140 USA

## Abstract

*Objective:* The objective of this study was to investigate the correlation of endometrial culture results with the clinical diagnosis of acute pelvic inflammatory disease (PID).

*Methods:* A total of 130 patients admitted with the clinical diagnosis of acute PID were prospectively enrolled in this study. Endometrial cultures by transcervical aspirate currette were obtained from all patients.

*Results:* Of 130 patients, 114 were discharged with a clinical diagnosis of PID. Of these 114 patients, 112 had positive endometrial cultures for pathogenic organisms. The correlation between endometrial culture results and the clinical diagnosis of acute PID was 98.2%. When patients with only mycoplasmas in the endometrial cavity were excluded, the correlation between endometrial culture results and the clinical diagnosis of acute PID was 93.8%.

*Conclusion:* These data demonstrate the exceedingly high degree of correlation between endometrial culture results and the clinical diagnosis of acute PID. Therefore, endometrial cultures may serve as a useful adjunct in the evaluation of patients with a clinical diagnosis of acute PID.


Infectious Diseases in Obstetrics and Gynecology 3:56–59 (1995)
© 1995 Wiley-Liss, Inc.

### Endometrial Cultures in Acute Pelvic Inflammatory Disease

**Soheil Amin-Hanjani and Ashwin Chatwani**

*Department of Obstetrics and Gynecology, Goddard Medical Associates, P.C., Brockton, MA (S.A.-H.), and Department of Obstetrics and Gynecology, Temple University School of Medicine, Philadelphia, PA (A.C.)*

ABSTRACT

***Objective:*** **The objective of this study was to investigate the correlation of endometrial culture results with the clinical diagnosis of acute pelvic inflammatory disease (PID).**

***Methods:*** **A total of 130 patients admitted with the clinical diagnosis of acute PID were prospectively enrolled in this study. Endometrial cultures by transcervical aspirate currette were obtained from all patients.**

***Results:*** **Of 130 patients, 114 were discharged with a clinical diagnosis of PID. Of these 114 patients, 112 had positive endometrial cultures for pathogenic organisms. The correlation between endometrial culture results and the clinical diagnosis of acute PID was 98.2%. When patients with only mycoplasmas in the endometrial cavity were excluded, the correlation between endometrial culture results and the clinical diagnosis of acute PID was 93.8%.**

***Conclusion:*** **These data demonstrate the exceedingly high degree of correlation between endometrial culture results and the clinical diagnosis of acute PID. Therefore, endometrial cultures may serve as a useful adjunct in the evaluation of patients with a clinical diagnosis of acute PID.**
© 1995 Wiley-Liss, Inc.

KEY WORDS

Pelvic infection, cervical cultures, mycoplasmas, pathogenic organisms

Acute pelvic inflammatory disease (PID) is due to an ascending spread of microorganisms from the vagina to the endometrium, fallopian tubes, and other pelvic organs. The polymicrobial etiology of acute PID is well established,[1] while the optimal site for culture of the microorganisms involved in this process continues to be controversial.[2] The recommended sites for culture are the fallopian tubes by laparoscopy and the cul-de-sac by culdocentesis. The endometrial cavity, as an intermediate organ in the development of acute PID would seem an ideal site to study microorganisms involved in the disease process. However, data on its correlation with clinical PID are lacking. Therefore, this study was undertaken to investigate the utility of endometrial cultures in the evaluation of women presenting with a presumptive diagnosis of acute PID.

### SUBJECTS

A total of 130 consecutive patients admitted to Temple University Hospital with a clinical diagnosis of acute PID were enrolled in this study. The diagnosis of acute PID was based on the presence of abdominal pain and tenderness, uterine tenderness, cervical motion tenderness, and adnexal tenderness. An additional requirement was an elevated temperature, an elevated sedimentation rate (>20 mm/h), the presence of a purulent vaginal discharge, or an elevated C-reactive protein (>0.0625 mg/dl).

Address correspondence/reprint requests to Dr. Ashwin Chatwani, Department of Obstetrics and Gynecology, Temple University Hospital, 3401 N. Broad Street, 7OPD, Philadelphia, PA 19140.

Received September 28, 1994

Accepted April 4, 1995

At the time of the patient's admission, the cervix was wiped with a sterile swab and endocervical cultures were obtained for *Chlamydia trachomatis* and *Neisseria gonorrhoeae*. Endometrial samples were taken using the transcervical aspirate curette (Pipelle®) under asceptic conditions and processed for *C. trachomatis* and *N. gonorrhoeae*. In addition, endometrial cultures were obtained for *Mycoplasma hominis*, *Ureaplasma urealyticum*, facultative aerobes, and anaerobes. For facultative aerobes, a port-a-cul medium was inoculated onto sheep blood agar plates, MacConkey agar plates, chocolate agar plates, Martin-Lewis agar plates, and V-agar plates. For anaerobes, the port-a-cul medium was inoculated onto CDC anaerobe blood agar, CDC anaerobe laked blood agar with kanamycin and vancomycin (BBL, Cockeysville, MD), and prereduced PRAS brain heart infusion broth (Adams Scientific, West Warwick, RI). The cultures for mycoplasmas were put into Shepard's 10B broth and transferred to the reference laboratory at −70°C on dry ice. The broth was inoculated at 37°C under atmospheric conditons. The growth of mycoplasmas was suggested by an alkaline shift due to urease activity by *U. urealyticum* or arginine hydrolysis by *M. hominis*. The broth cultures were incubated for 5 days before they were deemed positive or negative. For morphologic identification, the positive broth cultures were inoculated on A-8 agar plates just as the pH indicator began to turn. The A-8 agar plates were incubated under 5% carbon dioxide. *M. hominis* colonies were identified by their typical "fried egg" appearance and *U. urealyticum* by black colonies.

### RESULTS

A total of 130 patients were enrolled in the study. Their ages ranged from 17 to 38 years, with a mean (±SD) of 23.6 (± 4.6) years. The gravidity and parity were 2.2 ± 1.9 and 1.6 ± 1.2, respectively. Seventy-nine percent were black, 11% were white, and 10% were Hispanic. In 16 of the 130 patients, the diagnosis was changed after admission, and these 16 were excluded from the study (appendicitis in 2, ruptured ovarian cyst in 3, ovarian torsion in 1, lower urinary tract infection in 10). Of the remaining 114 patients, 112 had positive cultures for pathogenic organisms. *M. hominis* was the most common organism isolated from the endometrial cavity (71%). *U. urealyticum* was isolated from 44 patients (38.6%). Of 81 patients with mycoplasmas in their endometrial cavity, 76 grew other organisms as well. The correlation between positive endometrial cultures and the clinical diagnosis of acute PID was 98.2%. Considering mycoplasmas to be nonpathogenic and excluding them, we found the correlation between positive endometrial cultures and the clinical diagnosis of PID in the remaining 107 patients to be 93.8%. Sixty-two patients had positive cervical cultures for *N. gonorrhoeae*. Of these 62 patients, 54 also had positive endometrial cultures. The correlation between cervical and endometrial cultures for *N. gonorrhoeae* was 87%. The correlation for *C. trachomatis* was 89%. The results for the other organisms are presented in Table 1.

Of the 16 patients excluded from this study, the cultures of 10 patients grew *Mycoplasma* species from the endometrial cavity and the cultures of the remaining 6 patients showed no growth.

### DISCUSSION

Acute PID is polymicrobial in origin. In all the published reports, the incidence of positive cultures at various sites in the upper genital tract varies considerably in patients with acute PID. The positive cultures for *N. gonorrhoeae* have been reported to be between 39%[1] and 94%,[3] while those for *C. trachomatis* have been between 5%[2] and 47%.[4] The endocervix cannot be used for obtaining other aerobic and aneaerobic cultures since most ot these microorganisms may be found in the normal vaginal flora. The suggested alternate sites are the fallopian tube exudate and the culdocentesis aspirate.

The isolation of microorganisms from the upper genital tract (culdocentesis aspirate and fallopian tube exudate) has been considerably lower and has not always shown a good correlation with the clinical diagnosis. The incidence of positive cultures from the upper genital tract exudate has varied from 64%[5] to 90%.[6] Nevertheless, these 2 procedures continue to be recommended for diagnostic confirmation and microbiologic evaluation of acute PID. The lower incidence of positive cultures from fallopian tube exudate or culdocentesis aspirate may result from the difficulty in culturing certain organisms from pus. Or, these organisms may attach to and invade the epithelial cells and tend not to be present in the exudate at all.

It is well established that acute PID is an ascend-

**TABLE 1. Results of endometrial cultures**

| | Endometrial cultures (N = 114) | | Cervical cultures | | |
|---|---|---|---|---|---|
| Organisms | No. of positive | % Postive | No. of positive | % Positive | Cervical/endometrial correlation (%) |
| *Neiserria gonorrhoeae* | 54 | 47.4 | 62 | 54.4 | 87 |
| *Chlamydia trachomatis* | 16 | 14 | 18 | 15.8 | 89 |
| Nongonococcal/nonchlamydial organisms | | | | | |
| Aerobes | | | | | |
| *Escherichia coli* | 18 | 13.2 | | | |
| *Streptococcus* | | | | | |
| Group B | 12 | 10.5 | | | |
| Group D | 14 | 12.3 | | | |
| *Streptococcus* spp. | 17 | 15 | | | |
| *Staphylococcus* spp. | 10 | 8.8 | | | |
| *Actinomyces* spp. | 1 | 0.9 | | | |
| *Enterobacter* spp. | 3 | 2.7 | | | |
| *Pseudomonas* spp. | 1 | 0.9 | | | |
| Anaerobes | | | | | |
| *Peptostreptococcus* spp. | 5 | 4.4 | | | |
| *Fusobacterium* spp. | 1 | 0.9 | | | |
| *Gardnerella vaginalis* | 4 | 3.5 | | | |
| *Bacteroides* spp. | 34 | 29.8 | | | |
| *Mycoplasma hominis* | 81 | 71.1 | | | |
| *Ureaplasma urealyticum* | 44 | 38.6 | | | |

ing infection and that the endometrial cavity is an intermediate organ in the development of the disease process. Therefore, sampling the endometrial cavity would seem logical since it is easily accessible with the transcervical aspirate curette and the endometrial tissue itself can be readily obtained. Sweet et al.[2] reported that the endometrial cavity more closely resembles the fallopian tube exudate than the aspirate from culdocentesis. However, obtaining exudate from the fallopian tubes is more difficult as it requires the invasive procedure of laparoscopic examination. Culdocentesis, on the other hand, can be painful and may be associated with the inadvertent rupture of a tubo-ovarian abscess.

In our series, the incidence of positive endometrial cultures in patients discharged with a diagnosis of acute PID was 98.2%. There were only 2 patients in our study whose endometrial cultures were completely negative. It should be noted that the role of some microorganisms such as *Mycoplasma* species in the pathogenesis of acute PID is controversial and mycoplasmas may not play a pathogenic role in PID, as 10 of the patients excluded from our study were noted to be positive for these microorganisms. In the patients not excluded, 5 patients had only mycoplasmas in their endometrial cultures. The significance of this finding remains unclear.

No control group was available in this study for direct comparison. Although some early studies[7] have reported the endometrium to be a generally sterile environment in asymptomatic patients, more recent investigations[8] have reported the presence of bacteria in patients with no signs or symptoms of current or previous pelvic infection. However, the majority of bacteria isolated in these studies are not routinely seen in patients with acute PID. *Staphylococcus* and *Peptostreptococcus* species are the most commonly isolated bacteria in these reports.[8] Since *Staphylococcus* and *Peptostreptococcus* species were not the most commonly isolated organisms in our study, we believe that the positive cultures in this report truly reflect endometrial sampling and the microorganisms involved in acute PID.

Examining the microbiological milieu of the endometrium poses some clinical difficulty in that transcervical instrumentation may cause contamination,[9] which could not be totally excluded as a possibility in this study. All attempts were made to minimize contact with the cervix, and the cervix was cleansed with Betadine solution before attempting endometrial sampling.

As endometrial sampling is easy and accurate, endometrial cultures should be considered an adjunct in the evaluation of patients with a clinical diagnosis of acute pelvic infection.

### REFERENCES

1. Eschenbach DA, Buchanan T, Pollock HM, et al.: Polymicrobial etiology of acute pelvic inflammatory disease. N Engl J Med 293:166–171, 1975.
2. Sweet RL, Draper DL, Schachter J, et al.: Microbiology and pathogenesis of acute salpingitis as determined by laparoscopy: What is the appropriate site to sample? Am J Obstet Gynecol 138:985–990, 1980.
3. Moniff GRG, Welkoos Sl, Baer H, et al.: Cul-de-sac isolates from patients with endometritis, salpingitis, peritonitis and gonococcal endocervicitis. Am J Obstet Gynecol 126:158–163, 1976.
4. Osser S, Poersson K: Epidemiology and sero-diagnostic aspects of chlamydia salpingitis. Obstet Gynecol 138:985–990, 1980.
5. Paavonen J, Teisala K, Heinone PK, et al.: Microbiologic and histopathological findings in acute pelvic inflammatory disease. Br J Obstet Gynaecol 94:454–460, 1987.
6. Chow AW, Malkasian KL, Marshall JR, et al.: The bacteriology of acute pelvic inflammatory disease. Am J Obstet Gynecol 122:876–880, 1975.
7. Anshacher R, Boyson WA, Morris JA: Sterility of the uterine cavity. Am J Obstet Gynecol 99:394–396, 1967.
8. Hemsell DL, Obregon, VL, Heard MC, et al.: Endometrial bacteria in asymptomatic, non-pregnant women. J Reprod Med 34:872–874, 1989.
9. Grossman JH, Adams RL, Hierholzer WJ, et al.: Endometrial and vaginal cuff bacteria recovered at elective hysterectomy during a trial of antibiotic prophylaxis. Am J Obstet Gynecol 130:312–316, 1978.
